# Complete genome sequence of a new quadrivirus infecting a member of the genus *Thelonectria*

**DOI:** 10.1007/s00705-021-05353-y

**Published:** 2022-01-11

**Authors:** Tobias Lutz, Gitta Langer, Cornelia Heinze

**Affiliations:** 1 Institute of Plant Science and Microbiology, Molecular Phytopathology, University of Hamburg, Ohnhorststr. 18, 22609 Hamburg, Germany; 2 Nordwestdeutsche Forstliche Versuchsanstalt, Grätzelstr. 2, 37079 Göttingen, Germany

## Abstract

A novel dsRNA virus named “Thelonectria quadrivirus 1” (TQV1) was found in a member of the genus *Thelonectria* (*Ascomycota*), isolated from a root associated with stem collar necrosis of *Fraxinus excelsior L.* The complete genome of TQV1 is composed of four segments, each containing a single ORF on the positive sense RNA. The sequence of the 5´ (5´-(C/T)ACGAAAAA-3´) and 3´termini (5´AT(T/G)AGCAATG(T/C)GC(G/A)CG-3’) of dsRNA 1 (4876 bp), dsRNA 2 (4312 bp), dsRNA 3 (4158 bp), and dsRNA 4 (3933 bp) are conserved. Based on its genome organization and phylogenetic position, TQV1 is suggested to be a new member of the family *Quadriviridae*. This is the first report of a mycovirus infecting a member of the genus *Thelonectria.*

**Supplementary Information:**

The online version contains supplementary material available at 10.1007/s00705-021-05353-y.

The most widespread mycoviruses are viruses with a dsRNA genome. According to Kotta-Loizou et al. [1], dsRNA mycoviruses are arranged in seven families (*Totiviridae*, *Partitiviridae*, *Megabirnaviridae*, *Chrysoviridae*, *Quadriviridae*, *Endornaviridae* and *Reoviridae*) and one genus (*Botybirnavirus*). At present, the family *Quadriviridae* consists of the single genus *Quadrivirus*. Within this genus, only one member, Rosellinia necatrix quadrivirus 1-W1075, has been confirmed so far [2, 3], but several other viruses have been proposed to belong to the family *Quadriviridae*.

The quadrivirus genome consists of four dsRNA segments, which are packed in non-enveloped spherical particles, 45 nm in diameter. The particles are composed of the structural proteins P2 and P4 and enclose the RdRP, which is encoded on segment 3. Segment 1 encodes a hypothetical protein with unknown function. The dsRNA segments range in size from 3.5 to 5.0 kbp, comprising 16.8–17.1 kbp in total [3].

Members of the genus *Thelonectria* P. Chaverri & Salgado are widespread fungi that belong to the family *Nectriaceae* Tul. & C. Tul., phylum Ascomycota [4], that can exist in a cylindrocarpon-like asexual state. Typically, they maintain a saprophytic lifestyle; however, they can cause small cankers or root rot on their hosts. Fruiting bodies are mostly spread on the bark of diseased, dying or recently dead broadleaf host trees [5, 6]. To date, no virus has been described infecting a fungus of this genus.

## Provenance of the virus material

The *Thelonectria* strain NW-FVA-1901 (GenBank accession ID: OK161009) was isolated from a necrotic root associated with stem collar necrosis of *Fraxinus excelsior* L. It was collected in the Waldgehege Fahrenstedthof, mark 24860, Böklund, Abt. 3410a in Schleswig-Holstein, Germany. Isolation and identification as a member of the species *Thelonectria* was performed as described by Langer [7]. Mycelium was cultivated on potato-dextrose agar (PDA), from which virus-like particles were purified as described by Lutz et al. [8]. Nucleic acids were extracted from particles using a Double-RNA – Viral dsRNA Extraction Kit (iNtRON Biotechnology, Seongnam-Si, South Korea). Isolated dsRNA was subjected to next-generation sequencing. Libraries were prepared using a Nextera XT DNA Library Preparation Kit (Illumina Inc., San Diego, CA, USA) and sequenced on a NextSeq 2000 (Illumina Inc., San Diego, CA, USA) instrument at the Leibniz Institute DSMZ (Braunschweig, Germany) as paired-end reads (2×151). *De novo* assembly was performed and contigs were analyzed using Geneious Prime software (Biomatters, New Zealand, version 2021.2.2). The 5’ and 3’ termini of each segment were determined by the single-primer amplification technique (SPAT), using an oligonucleotide with a phosphorylated 5’ end and a 2’,3’-dideoxyC group (23ddC) at the 3’ end as a blocker to prevent self-ligation (5’-PO_4_-TCTCTTCGTGGGCTCTTGCG-23ddC-3’) [9]. Amplicons were cloned into pGEM®-T Vector (Promega Corporation, Madison, Wisconsin, USA) and sequenced. Nucleic acid sequences and ORFs were analyzed using SnapGene Viewer (GSL Biotech, San Diego, CA, USA, version 5.2.4) and BLAST on the NCBI website. Sequence alignments and phylogenetic analysis were performed using MEGA X (version 10.2.4). A bootstrap test was conducted with 1000 replicates for the construction of a maximum-likelihood tree using the Le and Gascuel model with amino acid frequencies and a gamma distribution of 5 (LG+G+F) [10, 11]. Figures were generated and edited using Inkscape (inkscape.org, version 1.1).

## Sequence properties

**Fig. 1:**
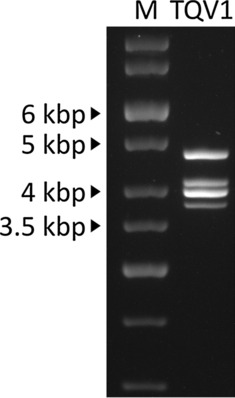
Agarose gel electrophoresis of dsRNA of TQV1 extracted from isolated virus-like particles from a member of the genus *Thelonectria*, isolate NW-FVA-1901. M, GeneRuler 1 kb DNA ladder (Thermo Fisher Scientific, Waltham, Massachusetts). Segment 1 to segment 4 of TQV1 range from around 4.9 kbp to 3.9 kbp.

**Fig. 2:**
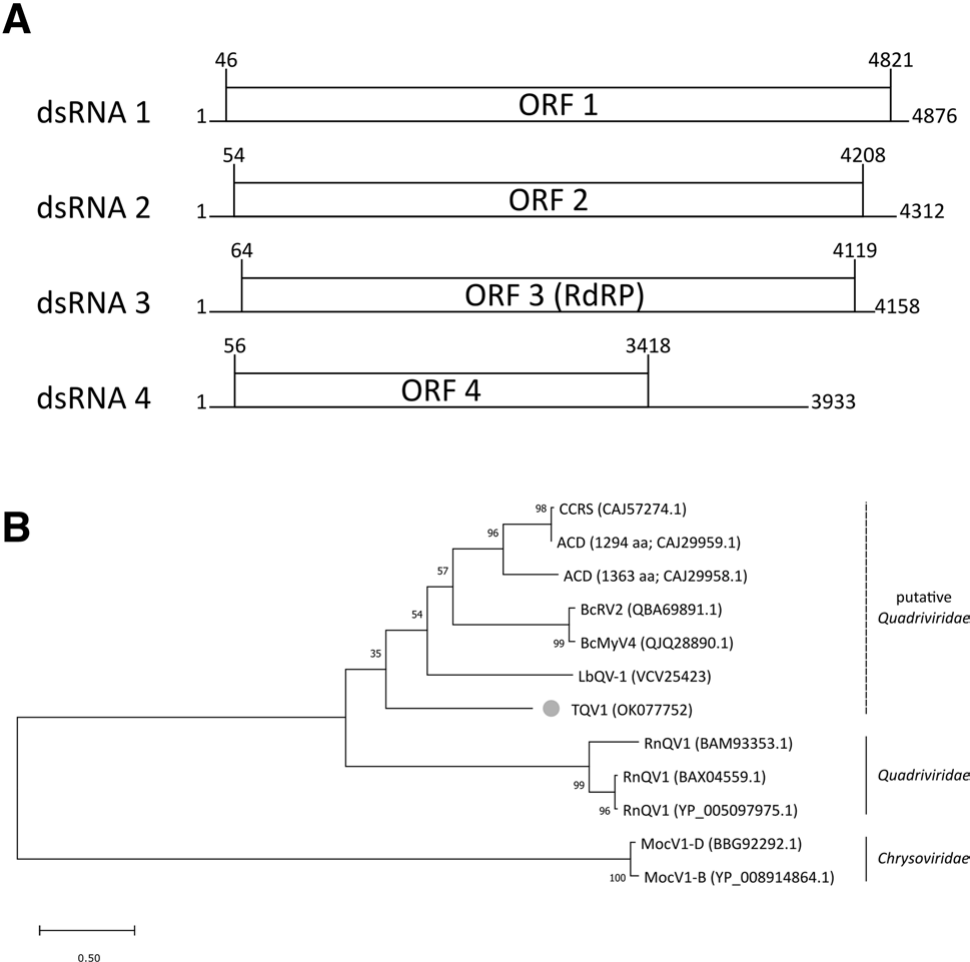
(**A**) Genome organization of Thelonectria quadrivirus 1 (TQV1). The dsRNA segments are displayed as horizontal lines with their respective UTRs at each terminus. ORFs are represented as boxes with start and stop codon positions indicated above the boxes. Note that the figure is not drawn to scale. (**B**) Maximum-likelihood tree of TQV1 and selected viruses with 1000 bootstrap replicates. Bootstrap values are displayed at the nodes. The scale bar (0.50) corresponds to the genetic distance. The grey dot indicates the new virus TQV1. The abbreviated names of viruses and dsRNA elements are as follows: CCRS, cherry chlorotic rusty spot associated totiviral-like dsRNA 4; ACD, Amasya cherry disease-associated mycovirus (1294 aa and 1363 aa); BcRV2, Botrytis cinerea RNA virus 2; BcMyV4, Botrytis cinerea mycovirus 4; LbQV-1, Leptosphaeria biglobosa quadrivirus 1; TQV1, Thelonectria quadrivirus 1; RnQV1, Rosellinia necatrix quadrivirus 1; MocV1-D/B, Magnaporthe oryzae chrysovirus 1 D/B

The complete genome sequence of TQV1 has been deposited in the GenBank database (accession ID: OK077750-OK077753). As it is typical for members of the family *Quadriviridae* [2, 12, 13], each of the four dsRNA segments contains a single ORF on the positive-sense RNA strand (Fig. 2A). The GC content of each segment ranges from 49 to 53%. The sequenced segments corresponded in number and size to the bands detected by agarose gel electrophoresis (Fig. 1), which showed bands ranging from 4.9 to 3.9 kbp.

Segment 1 is 4876 bp in length. Its ORF spans from nucleotide position 46 to 4821 and encodes a protein with 1591 aa and a calculated molecular weight of 176.21 kDa. A BLASTp search showed the highest degree of similarity (28.29% aa sequence identity with an E-value of 1e-95) to the hypothetical protein P1 of Botrytis cinerea mycovirus 4 (BcMyV4; accession ID: MN954886.1).

Segment 2 is 4312 bp in length. Its ORF spans from nucleotide position 54 to 4208 and encodes a protein with 1384 aa and a calculated molecular weight of 149.77 kDa. A BLASTp search revealed distant similarities to the structural protein P2 of BcMyV4 (accession ID: MN617035.1; E-value 6e-127; 26.17% aa sequence identity) and the putative capsid protein P2 of Leptosphaeria biglobosa quadrivirus 1 (LbQV-1; accession ID: VCV25422.1; E-value 4e-99; 26.08% aa sequence identity).

Segment 3 is 4158 bp in length. Its ORF spans from nucleotide position 64 to 4119 and encodes a protein with 1351 aa and a calculated molecular weight of 149.00 kDa. A BLASTp search showed similarities to RdRPs of nine confirmed or putative quadriviruses, with an E-value of 0.0. The highest amino acid sequence identity (43.33%) was shared with P3 of Amasya cherry disease-associated mycovirus (ACD; accession ID: CAJ29958.1), and the lowest value (35.28%) was shared with P3 of Rosellinia necatrix quadrivirus 1 (RnQV1; accession ID: BAM93353.1).

Segment 4 is 3933 bp in length. Its ORF spans from nucleotide position 56 to 3418 and encodes a protein with 1120 aa and a calculated molecular weight of 119.98 kDa. A BLASTp search revealed distant similarity (30.00% aa sequence identity) to the hypothetical protein P4 of BcMyV4 (accession ID: QJQ28881.1; E-value 4e-120) [14] as the highest value. The lowest aa sequence identity (26.34%; E-value 3e-19) was shared with the structural protein P4 of RnQV1 (accession ID: YP_005097973.1)

The 5’ UTRs of each segment range in length between 45 bp and 63 bp (dsRNA 1, 45 bp; dsRNA 2, 53 bp; dsRNA 3, 63 bp; dsRNA 4, 55 bp). Segments 2 and 4, which encode putative structural proteins, share 45.6% nt sequence identity at 5’termini of their UTRs. The 3’ UTRs of each segment range from 39 bp to 515 bp (dsRNA 1, 55 bp; dsRNA 2, 104 bp; dsRNA 3, 39 bp; dsRNA 4, 515 bps). All segments possess identical terminal nonamer sequences: 5’-(C/T)ACGAAAAA-3’ at the 5’ terminus and 5’-AT(T/G)AGCAATG(T/C)GC(G/A)CG-3’ at the 3’ terminus. Sequence alignments are shown in Supplementary Fig. S1 and Supplementary Fig. S2, respectively.

In total, the genome of TQV1 consists of 17.279 bp. To determine the taxonomic position of TQV1, a maximum-likelihood tree was constructed based on a BLASTp search of the sequence of the putative RdRP (P3) of TQV1. It was aligned with P3 sequences of confirmed and tentative members of the families *Quadriviridae* and *Chrysoviridae* (Fig. 2B). The phylogenetic analysis showed that TQV1 may represent a new clade between the clade including RnQV1 and the other viruses tentatively assigned to the family *Quadriviridae*. However, there is currently little sequence information about quadriviruses, and this lack of additional sequences correlates directly with the low bootstrap values observed in the tree. Therefore, the taxonomic position of TQV1 can only be assumed based on this phylogenetic analysis. Nonetheless, based on its genome organization and its phylogenetic position, TQV1 is suggested to be a new member of the family *Quadriviridae*.

## Supplementary Information

Below is the link to the electronic supplementary material.

Supplementary file1 (DOCX 19 KB)

Supplementary file2 (DOC 148 KB)

## Data Availability

The datasets generated and/or analyzed in the current study are available in the GenBank database (accession ID: OK077750-OK077753 and OK161009).
