# Supplementary material for: Complete genome sequence of a new quadrivirus infecting a member of the genus Thelonectria
Source: Arch Virol. 2022 Jan 11;167(2):691–4. doi: 10.1007/s00705-021-05353-y (PMC8843899; doi:10.1007/s00705-021-05353-y)
Supplement: Supplementary file 1 — Supplementary file1 (DOCX 19 KB) [file 705_2021_5353_MOESM1_ESM.docx]

>1901-RNA1-complete (4876 bp) OK077750

CACGAAAAAACTATAATAACGGTAACGATTGCAACATAGCTCAACATGATCACCGACACTTGTGAATTGATCAAGCGCAT

TACGGCTGCTAGGCAAAGCACCTACAAATCTCAGCTTGCAGCCGTTAAGATGAAGCACTCTACTCACGCGGATGTGAAAG

CTGAATTCAGCAGACACATCGCAATGGCTGCGGCTAAGATGGCGTCAAGCGACCCAACTAACATCATCGGGGACAGAAAG

ATCACCGTAACTGGTACGTTGACGAACTATGACACCATTGGGAGAGCTTGGCTCGATGACCCACTGGCCAAGAGACTACC

ATCTTTGGCACCGACTAACCTCTTGCCGGTTAAGCGAGATGACGAACTCAACGATGGTATGCTGACGGTCTGCGCTGACA

CCGAAGAAGCGCACGGATTCACTACTGACCCAGACGTAATGCACATCGCGCATGGCGTCCAAGCTGCCAACTTGCTATAC

GTGTGTGCTAGGCCCTCTGAGACAGCCATAGTGAATGAACCGATGAACGGAGAAGATAGCACCAACACCAGGGCTATGGC

GATCCTAACTTATGCATCGCTGGCCGCCAGGTATAGACGGCTGTTTCGTACTGGCAATCACATCCACGTCGAGCACGCAC

TAGCCACGGTCGCAACTTCACACGTGATCAAGCTTGAAGCCGATATGGCGCTCTCTGGGCGAACGTATGATATGATGTTG

GCAACTAAGAGCAACCGACTGAAGTTAGATACTGGAACGCGCTCCCAGAAGAGACTACTGTGCACTAACCACCTAGTGGC

AGATGCACTGAGCATTGCTGCCGGCGCTTTCCCAAAAATGTGGATCTCGCTGTTTGGTGACATAATGCCACCAAAACACA

CCGCAGCGACGCACGACCTAACAGGTGTGATGCGACTGGCGATATCACACTTGGCAGTCGCTAACACACCCGGTAACATG

GCAATGGCCATTGCCGAGTGCGCGGCAGGGTTTGGCAAGGTAGTCGCGACGTCGGACGAAGGGCAGCTTGAGTACTTTAC

ACCCACTATTGATTGTGGCCCAATCAGCAGCATGCGAGTCGATACTCGCACGAAACCTTTGCTGAATGCGGCCGAAGTTG

CTGCAGCACAAGCATCAGGAATCATCACTCTCGAGCTAGCGCTCACGAGGCTGCTGGTGACGCATGCGAGGCAGTTCAAC

TGCGAACGATACGAACGGTTTGAACACTACAAACAATTGATTGATGCCGGATTAGCTTCAGCAATCTCTATCAGGGACAA

ATCGTGCACCCTATACATAACAAACCTGCTTGCTACCCAGACGTACTGGAGCGTGTACGACATTGACACGTCTGCATGCA

ACGATGAAGAGCAACAGGCGCAAGGGTTTGGAATAACAGAGCACGACCTTGGTAGCGAGCCTAAAACACAACCAGGCCTA

ATCAACACCAGGGCCGCGCTCTCCCAGAACTCATGGGAGGCAACCGTCATAAGAGAAGCTTACACTAAGTCAAGTGACAG

TTCGAGGTACACACACTCGGTCAACACGGCACCGACAGAATACGCTGGAGGAACGTACATGCAAGAGGCGACGAAGCTAA

CTAACGTGACGACTGTCCGATCGACAGCAGGCGCGCTACTCAATGCTATTACATGGCTTGATACCAGTGCGCAGCACACA

GGCCAGCTGGCTCCAGTCATGTTAATAAGGCAGGGAGTAGCAATTGCTGATGAAGCAACAATGGTCGCGATGGCTCCGGA

ACTTGGACGATATGGCTACCAGCTACGACTGACATCGGACCACGTGCTTGGCGTCAACAATGCAAGGAGGGAATACGCAA

TTTTATCCAATGGTCAGCATCTAACAGCGGCGAACCTAGTGAAGATCTCGAAACCATTGAGCAGAAGAGTACAAAGTTCT

CCCACGATGGCAACGATCATAATGGCACTAGCTAGGTCCTACAATCAAGATACGTGGCTAACAGTTCAGCGCAAGCTCAA

GACGCTGCGGGTTGAACACGGGGCACAGTTCAAACCAGGCGAATGGCTCAAGCAGAAGGTCGATAAGACGGCAAACATTT

TGGGAAGACAAACGTATACGACGCAGTTAGGAAAGGTTTGGGGGGCGAGACCAACTATACCAAAGAGCTCATCGGTGGCG

AAGACTCTTGGGAAGCGTGGTGGCTTTAGCTCCAAGCAAATTATGTTAGACGCGCCCGCTACGCTGCACGACGATGGAGA

GGCAGCATGGCATCTGTGTGGTGACTTAGTCACTATGGGAGCTAGTGCATACCAGGCGGTCCACACACTGAGAGTTGCGG

GAGTCAACATTGGACAAGAGACACTCGCAGCACTAGCGAATTGGGCAATGGCGCACATCAAACCCGATTTACTCAAGTAC

TTTTGGGGCAGCGACCTCGCAAGGGCGGTGGAAGTGTACACAAGACCGCTAAGCAAGACTAACAAGGGGAGTACGACAAG

AGCTGCCTGGCATGGAGTCACAGGGATGATCGTGATGGCTGCTAAGCTATTAGTGATTAAGCAGCTTGATGAGTCAAACG

CTGATAGCCAGACGCCAGGGGCACTATTGCCTGCATTAGACCAGATAGCGACAAGCGCAACTACCCTGGAGAAAGTGGTC

TTGTATGCGCTTGTAGGGTGTGACGCTGGTTGGGCAACACCTGAGACAGTTGCACTGATGTACGCGAAAGCCGACACGCT

AGTGTTGCCATCTACCACAATCTCAGACGTCAAGCAGAGTGTGCTGAGACACAACACAATAGCAAGGCCATTAACGAAGC

TCATGAACTGTAAGGTTGGACGAAGTTGTTGTGTGACACATTGCGAGCAACTACTAGAGAGAACGCACAAGTGCATGAAG

TGCGGGAAGGTCAGAGAGTGCAGAGTGTGTAAGCAGTTGATGACACTGACACCAATGAGGGCACCAATGAGTAGCACACA

CGGTGTCAACAAAGCACAGACCTACGAACAAAGCGAGGACGGATCGGAGTATGCTGGTGAAACGCAGGCCGCGGAGGTGA

CGACTACACTAAAGGCCGGCAACTGGCAAGAGCAGAGTGCACAGAGCGCGAGCACGGCAGACGAAAAACTAAGGCAGGCA

AGTCGCGATATGATATCTCGTGCCTACCCAGAAATAGCAAATGAAGACTGGGTGGTAGCAGGAGGAAGTTATGGTCAAGA

CATAGATGAAGAAGACGAAATGATTAGCCCAGTGGACCCAGAACTAGTCAAGCCTTTTGAGCGGATGACAGACTGGGCTG

ACACGGTTCCCACAAATCAATCCATGGTTACAGGAGACCCGCTTATTGAACCAAGCAAGCAACTCACGCCACAGAACCTT

GACGTGGTAGCGAATAACGAGAGCGATGCTTGCGCACTCAGACTAGTTACTAGCTGGGCAGAGGAGGTTGAAGCGGTGGA

ACAAGAAGTAAACACGACATCCACGGCCATGGCGGCAGAACAATCACCAGAGCCTGAGCGAACTAGGGAGAAGCAGACGG

TAACGGCGACAAGTAAAGGAGACGGACCACAACTACTAACTGCTGACAGCCAAGTCAAACTAGCGCCGACAAGCCCCCTA

GGCACAGCAGGCATCGACTACGGAAGCGGATTGCGGTGTTTCAGGAACATGACCGACGGCCAGCACCCACCAGTGATACC

GGTGTCGGACACGACTAGGGCCAACATGACGTATTACGTTGAGCAGGATCTTCCTACCATCAAAAACAAGGCACGACGGA

GCCTCGATCGCGTGGCGCCAAGAGCTCTAGACACCTGTCAGCGGTACATCAAGTCCCAAACTATCGTGCCGTTGGTGGAA

GACTCAGACGTGCGGCCGAGCTTCACTACACTAATAAGGCGAGCAATGGCCGAGGGTCTGGAGGAACCGTACTTGTCAAC

AATTGAACCGCACGCAACGGTACCAAGACAATCGGAAGAACAAGTGTACTATGAGGTGACAGGTAATTGCGCTTGCCCGG

GATGCGAGACATACAGACGAATGGATGTTGCACGAATCAAAGACAGAACCACGATCGACGCGCCATTTTGGCACTATGAC

ATGGGCGAGTTGCGAGAACTGGATATACGGCTGAACGAAGACGCACACCATAGGTACGGTACAGTCAACGTCGTCGACTT

CCCAGGTGATACGCAGATAGCTGTATCCTGCTGGTTAGCGTGCATCACGGGATACACGTTCAAGTCAACGCTGACTACCG

ACACTATGAAAGCCTGGCTGATATCACTAGAAAGGTACAGAGAGTTCGCATGGTCTGAACGCGTCATCGAAGAAGGTAAG

TGGGGGAGGTTCGACGCAGCAGATCTCAAATTGGTACGGATAGCGAACAACCCGATGCACTTAGACATGCACCTTGGTGA

AGAGGATAGCATGAGGTTTAAGAGCTACCCTGACGTGCGGGAGCAGAAGTGGGGGTATGACAACTCACTATATGAAGTGA

AGAACGAAGTCAGTGATGACTTCAAATTCACAGAAGGATTAGTCAAACGGTTATTGCAGTACGTCAAAGAAATGTTGCCG

ACAGCCCAACCGCACAAGGCAGAGGATGATAAGATCAGAGTACTCAAGATCAACGGTGGACATTTCGGCGACATGAGTCA

CTACAGAATTGAGAGGGAGATATTTTTTATGCCGCTGCGCGAGAGGATCACGCTGGTGAGAGAACGGGCGTGTCTTAATA

ACCTCAAGAACCACAGACAACCAAGACACAAGACTGACCCAGTCATGCGGCGAATGCTGGATAACATCTGGGGCCCAGAA

GCCAAGGAGATATTATACTAGATCAGTAGCATGATCTAAGGACGAAAAGAAGAACAACACATTAGCAATGTGCGCG

>1901-RNA2-complete (4312 bp) OK077751

CACGAAAAAACACTTAATACGACAACGATTCCCAAGTGAAAGTAGTAACAACCATGGCCGAGCGCAACTCAGAGCAAGCC

ACGAAGACCAGTGACCAGTCAACCAAGGCAGAGCTGGATGCTAGGAACAGGGTTCTCAAATCCGACCTAGTCATCGACTC

TTTCTCGACGGTTTACCAGGCACCAAAGATCTTCAAGGATGTTCAGCAGGTAATCGACCACGTCACGCCTGGTTTCAATG

AGAACATGTGGGCACATCAGCAAACCTTTGACATTGATGAGGTTATACAACTGACAGGCACATTGACTGACTATGGCAAA

AACCAAGACGTTACCAGGTTTAACGGTGCTAGGTTTGGCATCGTTTCGGGTGCAAACCCAAATGCAGGTAACGTGACAAT

GCACAAGATCAATGACCTCGAAGTCGCTGTGGCTCATGTCAAAGTCTATGGGCACGCTCACGGTCAAGACAGCGATCAAA

ACGCCATGGAACAATACTTCAACCGTCCTCAGATGGATGAAGTCAATTCTGAGCTCAGGCGAGCTCTGAACCCCAAGCCG

GGTGAGATGCGCAAGTCTCTGTACCCCGCGAACACTGACCCTACGGGATGGGTATTCACGATCCTCGAGCGCTTGACTGC

GCACAACTCAGCCGTTAGGTACGACCCTAAGACTAGGAAGGTCATGACTGATGAAGAGCACTCTACGTCAAGCACAATGC

TCTCTTGGCCACTCGCACTTGAAGCAGCTGATGCAGGCGCCACCATTTTGGGTGGCTGGCATAAGCACGGCGACAGCGAG

GCACTTGACGCGAGATCTGCACTCAACACGGCCCTTGCAATGATGGCCGCATCTCAAGCCCGCATCGTGCAAGTCACAAT

CCACGATGCCGCTGCAGCAGTGACGTACAACACCTCCAACAACCCCATGGACATTGGACCAAGAGGCCAGGTTTTTGCGA

CTGACGCTCCTAATGCTGATGGCGGAAAGTATCATTACTACATCGAGCGCCAAGATGACAACAGAGTGTGGTTGCACACC

TGCGTAACATCACTTTTCCGCAATTGTGGTGTGCACACGGCCGGCAGCGATACAGAGGTGCCAATGGATGGATTCTGCTA

CTATGCCACTGAACTTCAAGACATGCAACCTGAGGCAAGATTCTTATTGAGCAAAGGACGAGGAAACGCAACTTTGGCAC

CATCCCACATCGGCTCAGTTGAGCAATTGTACGCTAAGTCTATTGGCAGAGACCGAATGCCGTATCTAGCCATAAACCGA

CCAGACGACACGAGCGATTGGCGGTTGAGGAAAAGAGAGCTAGTTGTAGCAGCTTTGGCTCTACTGAGAGGCTATGGCGT

CAGGAACGAGCAGTACTATCACGCTGCAACCAAGCTCGGCTCAGTGATTGGCGCAACGTACATACTTGGCGACCTCAATG

GCGCAATTGCTGTGCCAGCGGTAGAGCTTCACTTGCCAAGCATCATGACTTACGGCATTCAAAACTTGCGCATCACAAAC

GTGTACGGAGTTGCAGCACCACTAGACTTAGGCTTAGCGAGGAACTTTGTCAACGTCCCGATCCTTATTGGGATAACGAC

ATCATTGCTGTCTAGGATGTGGATTGCTTCACTAGTGGTGAACGCTGGAACTATGGACATCGGTTCAGGCACAAATGTGC

TGGATGCGGTGAAAGCACACAAGACTACAACGTTTACGCACCCAAACACGAAAGTGACGGACAGGCGCCCAATGACAGAA

CTGGCCGCTGATTCGTGGGTCCCAGGGAGGGCAGCCGCAGCATATCTCGCGAGTGCGGACGGACAGGGGCTGGACGAGCT

AGCATCAATTTTGGCTGGCTACGACGCCGAAGCGCCCTCTGAAACTGCCCTGATTCGCGTATCATACAGTGGTAGACAGC

GACCTGGCCAGCTGAACAACCTAACTGACGCCGTGACGTCGAACACTAGGGAAGAAATCATGCATTTCGTCAATGGCACA

ACAGTCAAGCAATGGGTCAAAATACCGAAAGCAATTGACTCAGTCCAGAACCCACTCATCGGTATTGGCGACACTGCGGC

ATTAATCACGCCAGACGCAAAGGCACCGATTAAAAAACTAAGGTGTGAACCCAACGCTCAAGGAGCAGAGGCGGGGACTG

GCTCCTTGGCAAGGACCAGAATTGCATTTGCAAACCAAGTTGGGACGAGCGAGCACGCAATCATGTCAGCACTAAGTGCA

GCGAAAATGGATCAGATCCAAACCACTAAGTGGCTGACAGATGGCGGAGCACAACCACACTCTGCTATGGTCATGCCGGC

CATGCTAGCCTACTTGGTTGGAACCCCACTTGGCAAAGTAACAGCAACAGGGACTCGGGATGAAGGCCAGCGGAACAGAT

TGACTGTCAGTGGGAGCCCACTCTTGTCGACTTTGCCTGGGATCGTCAGTATGATGACGGGGCTTAACATCAACAACACA

GATAGCGTAGCTCTACCATCATCGATTGCCACGTCCTCGGAAGCATTGAAACAAGCGGCGCTCAGCGGAGAACTACACGT

TGAGATGGTCACAGTTAACAACCCATCGAAGCAAGCCAGAATCGAGGGCAGAAGCAGACAAGCCTTGACAATCAGCCCAC

AACGTTCGATTACTAGACAGGAGCTCAACACACCGACCACGCACGGCGTTGCCTTTGATCCGACAGGACGCATGATTGGC

ATGATGGCACCGGTACTGTACAGCGTCGCTACTGAGTCGCGACCGGCAGTGTATACACAGCCAAACACTGACAGTGCTGC

TGGCAAGGTGGATGAGCTCGCACACGAGATCGAAGACATCGAAGCTCGGCACACGATCAAAGATGATGAGGAAGCACTGA

TGGCTACACCAACCTTTGAGCACTACAAGGGTGGGTTGGTAGAAACCGGCGGCCTAGATGGTTTTGAGATGGACACAATG

CCCTCCTTACTATCCCTGGGGGCACCGACGACTGCTGCCGAAGACGCCGAAGACACCACTGGCGGCACGACCGGAAAATT

GGAAATAATTGTCAACGCAGCGCCAACAGCGGTTCGACTAATGTCAGAACTACTGTTGACGGGTGTGCCAGTAGCGGCTC

AGAGAGCACTGCGAAAGCTCATGATTGACAGAGCGAACCGTGAGGGTGGTGTGGCTCTACGGGGAAAGCCGGATGTGCTC

AACAAGGCAATGAACAGGGCGGCACCGGATGCACCCGCGAACCTAAGCGACGTGATCAAGGACGCGACGGAGCTGGGCGT

GGTACTGCCGCCAAGCGCAGTCAGCGCACTTGGGATGGCAGCCTACATTTGTGATGGAATGAGCACGTTCCCAATAGAGC

TTAAGACGGCACTAGTGCATACACCTTCACTTGCATCGCTCCGTGAGTTTGAGCAATATGCCGAGGACCTGAGGCTAGGC

TGCAAGGCGGTGACAGACACTGATGCAATGGCCACCGCTTTAGCTGACTGGTTGCCATCAACTGACCCGTTGATCAAGGA

CCTAGTGCTTGAGACCATGCATTCAATTGCCATCAGGCTAAGTGACACTGAGCCTACACGCTGCTTCACTTACAGGATCA

AGGGTGATGCTGCGGTCAGACCAAGCCTACAGCAGTTCTTGGATATAATGGACGGTAAGGCCGATGCCACGGAAGACCTC

CCTTGCGAAACGCAGGTGCTCGAACCAAATGTGACTGATTTAGAACTATTGTTCTTGCCAGCTGTCGCAATCGAGGACAC

GGTGTTTAACGTGTGGCAGGTGGTTGGTAACATCACAGGTGCCACGGATGCAGCAACGGTTGAACTAGACTGGTATGAAA

AAGCATTCAGCCTCGGAGCACAGTTTGACACAGGTGAAGTGTACAATGGGATGGCTGCGACTAGGGAGGCTTTCGACAAC

CACAATCTGGGCCAAGTGAGTGATGCGAGGGCTCTTGCGTACTCGACAGACCACGAACTACACAGAGCCATCAACCAAGT

AGCAACTACACACCTCCTACATACCGGCAAATTGTGGGTGGCCTCTTGTGACTATGTCGACGCCGAAGATGATGCTTACA

AGAAGTACGAACGGGTGATGAGACCACTAATTAAGGAGATGATCGGCGGCATTGGCGGTGGGTACGATGTCAAGACCCAC

AGATCACACGAGCTAAGGTTCCTTAGACCGGCACCAAGGCAGTCATAAACTACTAAGCATGTTACGGAGGACCTTCGAGC

TAGCGATAATGGTCTAGTCCTCATCACACTAGACCTTAGATAAAGATGAACTACACATTAGCAATGTGCGCG

>1901-RNA3-complete (4158 bp) OK077752

TACGAAAAAACTAACAAACTACTCAAGTAACTCACGTAACTTCAAGGTCAACCAACACCAATCATGGCTGCTTCAGCTTT

TATCAACACGTACGGCGCATATGAGGAGGGGCAGCGATCGAGTGAGGAAGATGAAGTCAGAGCAGAAAAGACGCTCTTAC

CTACGATGACAGCGTTGTACGGTCGCAAGGTTAGGAAATCGACCAACCAATGGCAGTTGATGCCAGAAACGACTGAAGCG

TTAACGGCTCAATGGCTTAACCACGATGATGTCACTTCCGCACTACTAGCAAGCAAAGAACCAGAAATACTCAAAATCTG

TGACACGCTGGCATTGACAGGCAGGAGTGGAAACTTGGCATCCGGACCTAGCTACTCTTTAAGAAGTGGCGAGTTGTGCG

AGATCAACCAAGAAGCCGTTTACCAAAATTTTCGTGCAATGGTGTTTGAAGGCAGAGGACACAAGATGGGTATGGTGAAC

GAGATCACACCGCTGGTCACGACAGTGCCAAACACGGCTGAGCTCTATACTAACGTTGGTGCTACACTTGGAGTCATCAA

AAGGACGATTGGGCCAGTCGCGGCAATCACTGAACTCGATGCAATTACAAGGGCTCTTGAGAATGAGTTAACTAAAGCAG

TGAACATGCCGATGGCTACTAAGCGGCTGATCATCCACGTACTAACGCACGTCAATACTTGCGAAGAATCAATCCCTAAG

ATGTCAATCAGGTCGAGGAAGGAAGATGGCAACTATAACTCAACCGGTCTAGACACGATCCAACCACTATACCATAGCGT

ACTTGGTTTGAGAACGAGATCACAGTGCGTGACGACTCACAGGAACAACAGGGAGAGAATACTCCACTATGTCGGCTTAG

CTAAGAAAGCATTTGCCAAGCACCACGGATTAACTGGCACGAACACTGCTGAAGCCAGCGCCATAAGTAGAGTTACCACT

AGCAGAGGTGACGCAGACAGCCGAAGCGGAGCGACCGCGCCAGGAGAACTTGGCAAAGTAGATCAAATACTCACTGATGT

CCAGACCGTGTACTGGGCATGGAACGTTACGGGTTGGGTGGCAGCTACAAAGTGGAGCGCTGATTTGTGCGCTGTCTTGG

ACAGAGGCATACTAGACGAAGAACAAGGCGCGGCGCTAACAGAGTGGGTATTTTCGCGTTCGACATACCGGCGTATGATA

GGGTCAAATGCTATACAAGACGCAACTAGAGACGTGTCCGCGAGCAAGGTAACGCAAGCAGCTACAACGACTGTCAGATG

GCGACGAGAGCTCAATTGCATTATCCACGCACTTGATGATCTAGAATACGCCGTAGTCGCAGGAAGCGTCCCACTTCAGA

TCAGCAGTGCGTACTGGTCCATGCTTGACTACTTGCTAGAAGAAAGAGGCACCGGATACGGCAACTTGCCAGCACTACCA

AGGTACTCAATGGTTGGAAAAAAGATCTGTTCAGCACCCGCTTACGATGAAGGAAGTGTTAATCCACCAATTTTATACGC

ACAGGTAGGGTTAAGCGGAGGCGCCACTTATGCTAACTTGGCACCAGCAAGCAGAGCTAACGGTATACTGCACAGCATTA

ACGCTGCGGACGCAAAAGATGGCAAACAACGGATCCGGACGGCGAGTGAGACCGCTGACATCTACACGGTAATGAATGGT

GATAACCTGTTATCATACTTGGTGCCGTCATCAATTAAGGGAACGGGAATCAACACGAGCTACGTGTACTTGGCTGGCAT

TCATTTTAGAGAAGACCAGTTGAAATACCCTGTCCCTCTACTTGAATTCATGAATGAATTCACAGATTATGCAGGACCAG

AGTATCAAACAGGCGAGGTCAATGACACGAAGCGGAAGACAACAGGGGTCAAGTTGAGACTCAAAGCGGATCCAGCACAC

ACTCAACAGCACCACAAGAACACGAGCTTCATACAGGTACTAGCAATATGTGGGCATGCATGGGCGCCGTGTTTGGACAC

GATAGCTAACTGGGATGACGCCAACCTAACATTTGCTACTGCATTGTTGATCGCTATGGCAGCATTACCACCAGAGTTGT

TTGCGTTGATGAAGCACTGGCGTGGATGGCGGGCGACATCAATGGCGGAGTACAGAGATTACGCTAAAAGGTTATCAGTG

AAGATGAAGGCACTGGATAACCAAGTCAGTATAGGCGACTATGTGCTAGACCTGTCACCACTCTTCGAGTGGGAAGTGCT

TCCGCACAGGGCAGTAGAGCACGGAGACATGAAAGGTGAGATCCTAGAGAGAAGGGATAAGAACCTACAAGTCAAATTGA

CGGCCCAGCAACTTAAACGTGACGTGACAGACGTATTTAAAGACGTGGCGGCCAAACTAGATGCTAGGACGAGAGAAGGC

AGTACTAGCCCTTTATACCAGGCATGGGAGGATTTCGTCGAAAATAGAGTCAACGCGACGCCCTCCGGGTCAGCTTTCAC

TGCTGATGAGCTCTTCATCAAAGCACGAGCCAAGCTCAAGGAGATAGGAGTTAAAGACCTGACGAAGACACAAGTGATGG

CCGCGATGCCAGACGTGCTAGCACTGGATGATTTGCTTAAGCGGGAACCAGAATTACTGGCACAAACCTCGTGGAAATAT

GAGTGGGGCAAGACCAGAGCACTATTTGCGGCGGCAATGGAGCACTGGGCTGTATCAGCGTTCGCTTTCGCTAAAATAGA

GGAATACATGCCAGACGATTGTCCTATCGGAAAAGCTGCTGACGCAGACCGAGTGTGTAAGCGAGTGATGGAACTAACGA

AAGAAGGGGTAGTGGCGTGCATCGATGCTGCAAACTTCAACATTTTGCACAGTCACGAGGTCATGGCCATGATCCACGAT

GTGTATGCTAGCGTTATGGGCAATAGAATATCGCCCGAGCAGCATAAAGCAATCAAGTGGCTACGGAGCTGCGAGCTCAA

CCAAAAAGTGATAGTCAGCACTAGCACTGTGGGTGACGAGCTAATCGCTAGAGGGCACTCTGAGGGGTGGATTAAAACAC

GAGAACTAGCTGACTCACGCACGGTAGAAGTGGCCGACCTGCACGGCGGAATGTTCTCGGGAGTGCGAATCACCATGCTA

GTAAACACGATACTCAACAGAGCTTATTATAGATATGCAGCTAGGAGTATAGGGATTAAACCACGGGCGCTACATTCCGG

TGATGATGTATACGCAGTTTTCTCTTCATATGCTGAAGCATATCAGATGAAGGGTGCCCTCCAGGCCATAGGCTACACTC

TGCAGCTGGCTAAGTGTTTCGTAGAAGGCGTCAAAGAGTTTTTGCGAATATCTCACAAGAACGCTAATACCACACAGTAC

CTTTCGAGATCAGCTGCTACAGCTGTCCATGGCAGAGTAGAAGCGGATAACCCAACAGATTTTGTGGCCACAGTTAACGC

AGTACTAAGGAGAGGTGCCGAACTAATTGTGCGCCACGCAGTCAAAGCACCCATAGCAGACTTACTGGTTGCTCAGACCA

GAAGCGTGTGCGCACGGTGGGGTGTAGCCAGGTATGCGTGGAACGCATTTCTAGCCCTACCAAAAATATTCGGTGGCTCG

TCAGAGGTAGCCGATCTGTCAGGGGCATGGTCAGGGATGACAATTGCAAGAACAGCCGAAGCACGAGGTGATGTAGTGCC

TAAGCTGGCAGACTTACCAGGCGTTGCCACTACGGCTCGCAAGTTACTGGAGGAACTCAAAGTGCGCAAGTTCCACAGAA

GAGTGAGGGAGGCGATAGCAGCGGCTATAGCACCAAAAGGCGTAGTAATGAACTATGGTATAGCACTAAGATGGATGACA

CGCACTGACGTAGAAAAACTACGTAATGTGGCGGGTACTTTGAAACATGTCACACAGGGTAGAGAATTCATACTAAGTAA

AGCTGCAGGCCTGTTTAACACACTGGCACTTAATGAACAATACTGGGGGGACTTGGCACCAATGCTAAGGGGCATACACT

CGAATTGGCATGGGCAAGTACTGGCATGTGCTTTAGCACCAGTGAGCGATGTCATAGACAACACAACAACCAAGCATGAC

TTAGATGACTGGATCTCTTCGGGGAGTCACAGGTTGTAGAAAAACAACAATGAACACATTGAATGAGCAATGCGCACG

>1901-RNA4-complete (3933 bp) OK077753

TACGAAAAATAACAAATTACCTAACAAACGCAAGACAGCATTCAACACGACCAACATGGAGAAGTTAACTGCACTAGCTG

GAGCACTCTATCCTGAGTCTGAGGCGTCCAAACAGCGCAAACAAGATGGCGGGACTAACACTGCACTCTTCAAGTACAAT

GAGGAGATGCACACGTTTGAGAAGGCTGTGAGCGACATCGCAAGTAAGATTGTCACCTCGATGAACATTTGCCCTGAGGT

TGATCTAGCCTCCATGCAGTACGACAACTGGCCTGCAATTGCTAAGATCAACAGAGGCTGGGTTGAATCTGAACTCCAGG

CAGTTGCAAAGGGCGAACATGGGGGAATGGCTCGCTACTTGGACAAGATGAGCAACTATCTCACTGATGCAGCCAAGACC

GACATCAACGCCCTGACTTATCCGGGTGACCCGCCAATTGACTATGAACAAGCTCACGCTAGCGAGCTCAAGGCGCTATC

TCAGGATGCCCCAGTCGTGATCGACGCGCTTATGCGCGCCAGACACGTCGCGACGAGCAACGCAGCGGCCAGGGTGGCTT

ATGCAGTCAATGAGCCCCTAATCTTCAAGGTCGGCAGACTGCACGCTAAAAGCACGATCAGTGCAGCGGTGCGCTCGAGT

GGCGAGCAGGTCGAAGGAGGGATGAATTCGACGGGCAGGACTGAGTACGATCTGGTAGTTGCATATGAGGCACTCAAGGT

TAGCATTGTCGGTGACGTCGCAAGAGCGCCGGAGACAGAAGGCCGCGCTGTTGGTGACAACGCGATGCTTGCTCAGCTGT

ACGCAGGGCGAGCGCTTACTGGTCAAGTAGCTCCAGGTGCCTATGATTACATGAAGGAAGCTGAGGCACTGGCACTCTTC

GACCAAGGCAGTGTGTCCACACCAGTCGCGGCAGCCTCAGTCATGCTGACTACACTCTATGGCTGGTTGTTGCACATGCG

CGCCGTTGGCGCTAAGGCAAATGAAAAACGGAGCTTTAAGGACTGGGTCGAAGCTTCGACGTACATCGCGAAACGTGGCT

TCTGGGACAATGAGATCGTGTCGGGATTATCACTGGCAGATGCTGAGCTAGCTGAATTGACGCCAGAGGCAAGCATCCAA

CACACCAAGTGGTACAGAGATGAAGAGGGTAGACTCGCGACTGTAGGCTGCTACGTTCACCAGCTGAGTACACAACAAGG

CAACGGCGCAGCACCTGGTATCAGGAGTGGCCACTACTTGCTGACGATCCCGACCTGGCATGCAACACAGCAACCGCTCA

CTGCGGCTGCTCTCGCGATGACTTATCAGCGCCCACGACTAGCTGGGGCCGACGGACACTTCCAGCATATGGCACTGGCC

AATGCTTGTAATGTCAGCTATGTGGCAGGTGTTGGTGCATCAGCTGGCCACTTACAGAGTGGCTGGAGCTCTTCAGTTGC

GTTTGGTGCAACCGAAATCCTCACGCTGATGGGGGCTCTAGTGAAGCACAATCTGACGCTGTCGCAGAACATCGTCTCAC

AGGTCACGTATTCACTTGCCTCTTTGTTCGCACCAGCAGCCAGATCGCACCACGACTACACGAACGTCTTAGGGGCAGGC

GTATTGCGCCTAGCACACCCATTCTGCGAACCGGCAGCAGACAAGTATTTCAACACAAAAACAGACAGAGCGCAAGTGGA

CGTCGACGTGACAGCAGAGGCGCTGGGAATGCAATTCACGAGCAACCTCGCAACGATGATCTCTGTGATGGGCACAGGGA

GATCCCTTGGTGAGATCGCTATCGCCCAAAGAGGCACTGAAGTCCTAGATGACGGTGACGTGACAAGGTTACAGCTTGAC

CATGCAGCGATGCTGATCAATCTAGGGCTGAGCGATGAACCTATCGAGAGCATCGCCAAGGCCCTCAAGACTGTAGGGGC

GAGTAGCACAGTCAGTACAGAGCTGGGCGCTATCAACCTTGCGGCAATGGGGGCTAAGCACATCAACGCCGCCGCTTCGG

GCTACCTGTGGCCGGTAACTGCAGTGACTCAATCACCCGGGTGTGCTTACAAGCGTGAGCACAACCGAGGAGCATTAACG

AGAGTCAAGTATAGAGATGGTGAATATCACGAGGATGCAGTAGAGGAAATCATGATTCACGGCCCAGGTAACGTGGCGAC

CATCAATGGAGCACACGCGACAACACGGGCAGTCGAGTACACAGCCCACGTCACTGAAGCTAGGCAAATGCAGCACACGG

CGCTGGAAGTATTCGCTGAGAAAATGTGGAAGACGAAACCCGCACTATTGTTCGCAGCGATCAAAGCTCTGGAGCGGACT

AACACTGTGATCAATGGCAAGCCGAACCAGTTGATTGAGCTCAAGCCACAGCTTGCCAAAAACTTGCTAACGCAGAGTTC

AGCTGATGATCAAGCACTCGTGTCGAACATGGACAAAGACGTCAAGGTTGGGGCTGAAGGCACTAAGATGAAAGACTGGA

TTCAGATGATCAGGGCAGAGAACAAAAGGACTGAAGCATACGAAAGGAACTCGGCCGCTATGGGAGACACAGCCGACAAG

CAGGCGCTACTACTAGCAGCTGCTGTCGTGGTCAAGATGATGCTGACGGCAATGGATGCGAGGACCACGTTCAGAGAGCA

GCACCCAATAAGGCAGGCAATTGCTGTCGGGTGGTGGCACGGCGCATATGTCGAGAGTGCAGCTAAGTGGTATGCGAAAA

ACCCCGACGGAGGATGGCGGCAGGCGATCAACAAGGGACATGCAGCTCTAACGACGGTATCGCTCAACCAGATTGAGAAT

TGCACGGGCACATGTGCGTTCAGTACCCTCTTGACCGAGCGAGCGATCCGCATAATAATCGCGGACGCTTCAGGGAGGTG

GACCACATTAGGACGAACCGTCGCTGGGGGAGCCGCGGTGCCTGCAGCGCTACGGGGATGGCAACTGAAGAGAGCAGATC

TTGTCAACGGCCTAGAGGGTAGGCAGCAAACCATTATCGACGTAGTAAGCAATGCAGCGGAGGAGAGCGACGCACTGATG

CTTAAGTTGAGTACCACCGCAGCTAGGTCAACTCAGGGGAGCGTCGTCGATACAAAGCGAGGCAAGCTCAAGAAGGGAGT

AGATGCCATAAGCGTCGTCACTTTTCTTGATGCCCAACGGGTTAGATTAGTAAGGTCGTACTACTCAGGAGGTGAGCATG

GCGCCAAAGACAGGCTACTACTGCACAACCTCGATAGAACAGAAGCGGCAAAGGCACTACCAGTAGTGTCAACGACCATG

CAACAAAGTGCCGCCGCAGTTGCGCAGTTGACAGCACAAATTAACGCACTTGGGAACGGGGGTACGTTCACTGATGCCGC

TGAAGCAGGACAACAAGTGCGAACTGGTCTGGCAATAGCAGGCTCGTCAATGTCATGAACCAAGGAACATAGTAGGTGGG

TGATAGCATGGCGGTCGGGCGTTATTTTCACTTGTGTGGGCGTTAGGGCGCAACGACGGATGTATTGACGGACTTTTGAC

GCCAACGAGGCTGACAGGCATTCGAACTTAAACTAAGGTACCAAAGGGTGGCGGCATGGAGGGTGCCACAGAGTAGTGCG

GACCAAGGACTGGCAAGCCGAGGATGTACCATAGCGGTCAGTAAACGATGGAGCGTGAACAAAGTGGCACGGATAAGGCG

TTAGGGATAGTATATCATTGGGTGGATGGAATTAACGGATCGACAAGGATGATGACTGCGCAAACCAATCTCAACAAAGC

ATAACACAAAACAAACTTTTCTAAAACAAACCAAAGCAAACAAAAACAACTAACAGACTGGCGGCTAAGACGGGATAGCC

TAGTTGAACACAGTACAGAGCGGAGCAGCTAAAACGCTGCAGGGCCAACGTGACTGTGGTGCCAGCGGGAACTACACATG

AGCAATGTGCGCG
