# Supplementary material for: Complete genome sequence of a new quadrivirus infecting a member of the genus Thelonectria
Source: Arch Virol. 2022 Jan 11;167(2):691–4. doi: 10.1007/s00705-021-05353-y (PMC8843899; doi:10.1007/s00705-021-05353-y)
Supplement: Supplementary file 2 — Supplementary file2 (DOC 148 KB) [file 705_2021_5353_MOESM2_ESM.doc]

Complete genome sequence of a new quadrivirus infecting a member of the genus *Thelonectria*

Tobias Lutz^1^

Gitta Langer^2^

Cornelia Heinze^1^

^1^University of Hamburg, Institute of Plant Science and Microbiology, Molecular Phytopathology

Ohnhorststr. 18, 22609 Hamburg, Germany

^2^Nordwestdeutsche Forstliche Versuchsanstalt

Grätzelstr. 2, 37079 Göttingen, Germany

Corresponding author

cornelia.heinze@uni-hamburg.de

Supplement


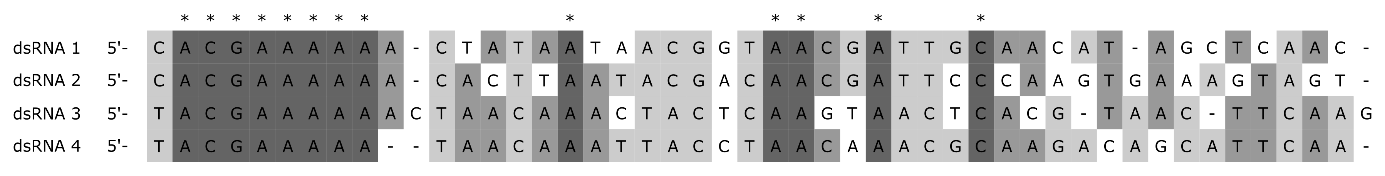


Supplemental Fig. 1: Alignment of the 5’-terminus nonamer sequences of all segments of TQV1. Conserved sites are highlighted in dark grey and with asterisks.


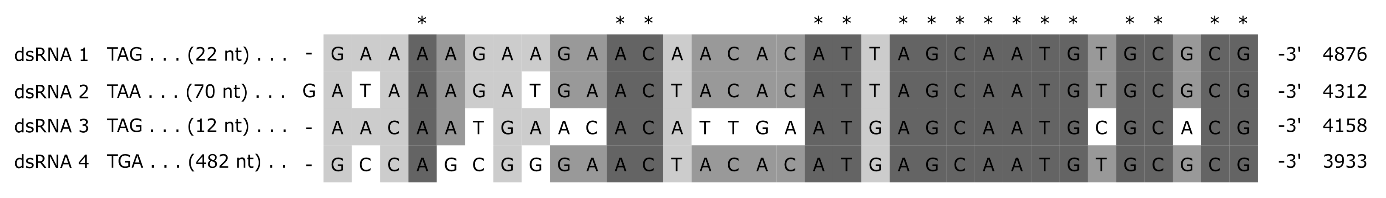


Supplemental Fig. 2: Alignment of the 3’-terminus nonamer sequences of all segments of TQV1. Conserved sites are highlighted in dark grey and asterisks.

The fungal strain NW-FVA1901 was identified based on a BLASTn search of ITS on the NCBI GenBank (www.ncbi.nlm.nih.gov) database on Nov 19th 2021. The search conducted revealed close affinities to the type strains of *Thelonectria olida* (CBS 215.67, MW827640.1, 98.99 % identity, 89 % coverage) and *T. truncata* (G.J.S.04-357, JQ403319.1, 98.82 % identity, 91 % coverage). The ITS sequence of NW-FVA1901 differs in five and seven nucleotides to these strains, respectively. The strain therefore belongs to the *Thelonectria veuillotiana*-species complex, more precisely to the clade contained of *T. olida* and *T. truncata* [1].

[1] Salgado-Salazar C, Rossman AY, Chaverri P (2016) The genus Thelonectria (Nectriaceae, Hypocreales, Ascomycota) and closely related species with cylindrocarpon-like asexual states. Fungal Divers 80: 411-455. doi:10.1007/s13225-016-0365-x
